# Supplementary material for: Chronic exposure to TNF reprograms cell signaling pathways in fibroblast-like synoviocytes by establishing long-term inflammatory memory
Source: Sci Rep. 2020 Nov 20;10:20297. doi: 10.1038/s41598-020-77380-9 (PMC7679373; doi:10.1038/s41598-020-77380-9)
Supplement: Supplementary file 1 — Supplementary Information. [file 41598_2020_77380_MOESM1_ESM.pdf]

Chronic exposure to TNF reprograms cell signaling pathways in fibroblast-like synoviocytes by establishing long-term inflammatory memory

Umesh Gangishetti Ph.D.,[1,2] Sergio Ramirez-Perez Ph.D.,[1,2] Kyle Jones BS,[1,2] Abul Arif Ph.D.,[1,2] Hicham Drissi Ph.D.[1,2,3] and Pallavi Bhattaram Ph.D. [1,2*]

[1]Department of Orthopaedics, [2]Department of Cell Biology, Emory University School of Medicine, Atlanta, GA 30322, USA

[3]Atlanta VA medical Center, 1670 Clairmont Rd, Decatur, GA 30033

*Correspondence:

Pallavi Bhattaram (pallavi.bhattaram@emory.edu)

Supplementary Material

Supplementary Figure 1. Scatter plots of RNA-seq data. Averaged normalized intensities from various experimental groups are presented. Green lines indicate 2-fold change.

Supplementary Figure 2. TNF expression levels in human and mouse FLS. (**A**) Averaged RPKM values of transgenic human *TNF* and endogenous mouse *Tnf* in RNA-seq from 11-week old wild type and Tg197 mice. (B) Averaged RPKM values of *TNF* gene expression in RNA-seq from normal and RAFLS.

Table S1. Experimental details and run accession numbers from NCBI-SRA database of the samples analyzed in this study.

Table S2. Expression levels (RPKM) of mSAGs in FLS from wild type control and TNF transgenic mouse Tg197.

Table S3. Expression levels (RPKM) of mSRGs in FLS from wild type control and TNF transgenic mouse Tg197.

Table S4. Expression level (RPKM) of SAGs and SRGs in human RA-FLS treated with TNF for 8h and 24h.

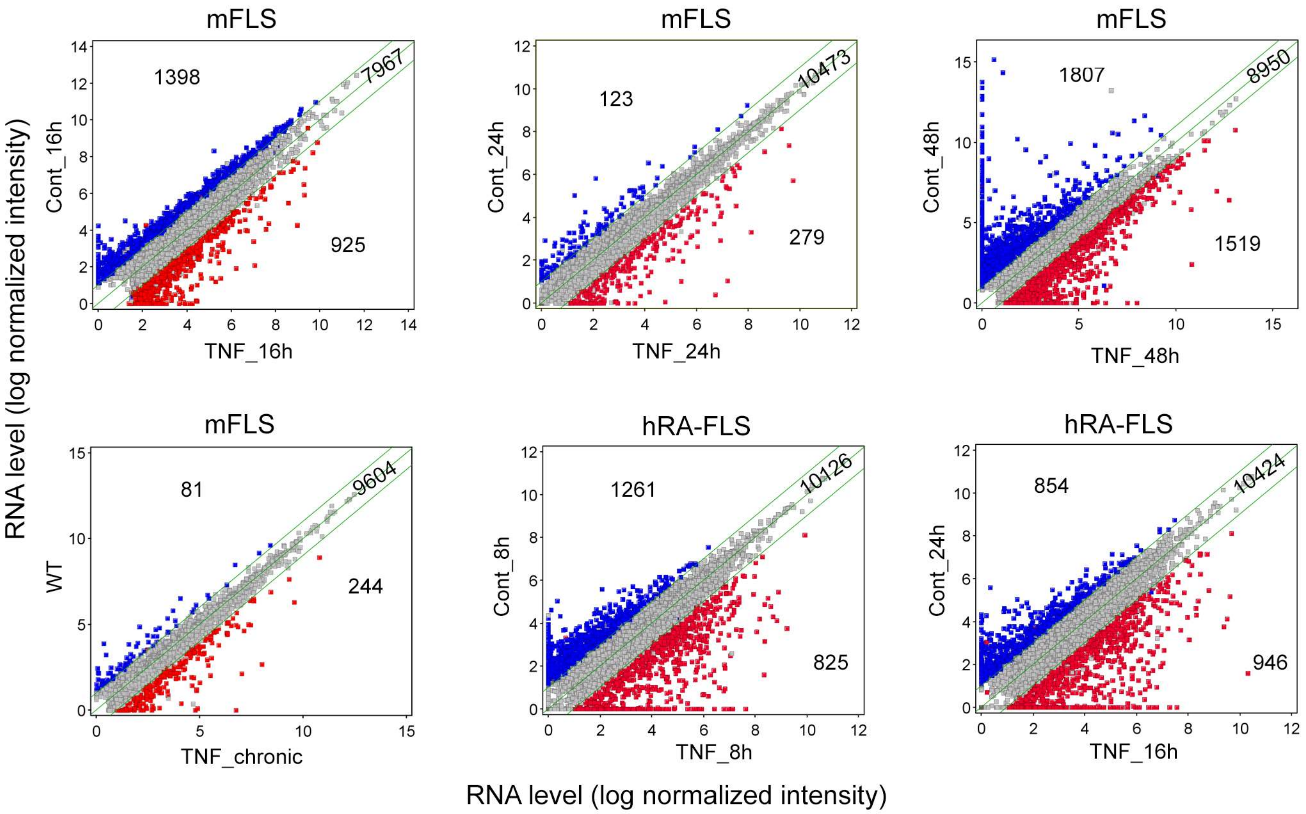
mFLS
1398
7967
925
Cont_16h
TNF_16h
mFLS
123
10473
279
Cont_24h
TNF_24h
mFLS
1807
8950
1519
Cont_48h
TNF_48h
mFLS
81
9604
244
WT
TNF_chronic
hRA-FLS
1261
10126
825
Cont_8h
TNF_8h
hRA-FLS
854
10424
946
Cont_24h
TNF_16h
RNA level (log normalized intensity)
RNA level (log normalized intensity)

Figure S1

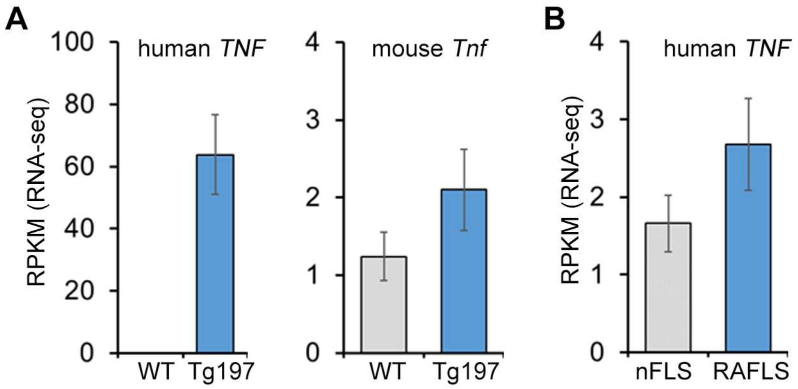
A
human TNF
RPKM (RNA-seq)
100
80
60
40
20
0
WT
Tg197
mouse Tnf
4
3
2
1
0
WT
Tg197
B
human TNF
RPKM (RNA-seq)
4
3
2
1
0
nFLS
RAFLS

Figure S2

Table S1. Experimental details and run accession numbers from NCBI-SRA database of the samples

RNA-seq mouse FLS

Sample name	phenotype	age	FLS passage	Run Accession	Project number
WT-1	healthy	11 weeks	4-5	SRR5279907	PRJNA376646
WT-2	healthy	11 weeks	4-5	SRR5279908	PRJNA376646
WT-3	healthy	11 weeks	4-5	SRR5279909	PRJNA376646
TNF chronic-1	inflamed	11 weeks	4-5	SRR5279910	PRJNA376646
TNF chronic-2	inflamed	11 weeks	4-5	SRR5279911	PRJNA376646
TNF chronic-3	inflamed	11 weeks	4-5	SRR5279912	PRJNA376646
Cont 24h-1	healthy	8 weeks	4-5	SRR5279898	PRJNA376646
Cont 24h-2	healthy	8 weeks	4-5	SRR5279899	PRJNA376646
Cont 24h-3	healthy	8 weeks	4-5	SRR5279900	PRJNA376646
TNF 24h-1	healthy	8 weeks	4-5	SRR5279904	PRJNA376646
TNF 24h-2	healthy	8 weeks	4-5	SRR5279905	PRJNA376646
TNF24h-3	healthy	8 weeks	4-5	SRR5279906	PRJNA376646
Cont 48h-1	healthy	13 weeks	3	ERR2562029	PRJEB25978
Cont 48h-2	healthy	13 weeks	3	ERR2562031	PRJEB25978
TNF 48h-1	healthy	13 weeks	3	ERR2561961	PRJEB25978
TNF 48h-2	healthy	13 weeks	3	ERR2561962	PRJEB25978
Cont 16h-1	healthy	8 weeks	4	SAMN15430181	PRJNA643827
Cont 16h-2	healthy	8 weeks	4	SAMN15430182	PRJNA643827
TNF 16h-1	healthy	8 weeks	4	SAMN15430183	PRJNA643827
TNF 16h-2	healthy	8 weeks	4	SAMN15430184	PRJNA643827
**DNase-seq mouse FLS**					
Cont 1	healthy	13 weeks	3	ERR2561546	PRJEB25978
Cont 2	healthy	13 weeks	3	ERR2561547	PRJEB25978
chronic TNF-1	inflamed	13 weeks	3	ERR2561540	PRJEB25978
chronic TNF-1	inflamed	13 weeks	3	ERR2561541	PRJEB25978
**ATAC-seq mouse FLS**					
Cont_16h	healthy	8 weeks	4	SAMN15430179	PRJNA643827
TNF_16h	healthy	8 weeks	4	SAMN15430180	PRJNA643827
**RNA-seq human FLS**					
RA FLS-1	RA	unspecified	4-5	SRR11074910	PRJNA606298
RA FLS-2	RA	unspecified	4-5	SRR11074917	PRJNA606298
RA FLS TNF 24h-1	RA	unspecified	4-5	SRR11074915	PRJNA606298
RA FLS TNF 24h-2	RA	unspecified	4-5	SRR11074922	PRJNA606298
RA FLS TNF 8h-1	RA	unspecified	4-5	SRR11074913	PRJNA606298
RA FLS TNF 8h-2	RA	unspecified	4-5	SRR11074920	PRJNA606298
**ATAC-seq human FLS**					
RA FLS-1	RA	unspecified	4-6	SRR8758524	PRJNA528326
RA FLS TNF 24h	RA	unspecified	4-6	SRR8758527	PRJNA528326
RA FLS TNF 72h	RA	unspecified	4-6	SRR8758526	PRJNA528326

Table S2. Expression levels (RPKM) of mSAGs in FLS from wildtype control and TNF transgenic mouse Tg197

Gene Symb	**WT-1**	**WT-2**	**WT-3**	**TNF_chronic-1**	**TNF_chronic2-1**	**TNF_chronic-3**	**p-value(T-test)**
Abca3	1.81	1.54	1.62	3.82	2.80	3.55	0.01
AC125099..	0.00	0.00	0.00	4.13	5.25	5.08	0.00
Acp5	1.23	0.61	0.51	8.70	2.96	5.69	0.04
Adam8	5.53	2.20	1.28	11.29	6.24	8.80	0.01
Adamts14	1.03	1.04	0.68	2.20	4.43	3.44	0.03
Adamts7	1.76	1.41	1.25	8.26	5.95	4.84	0.01
Agtr1a	13.24	6.95	12.06	34.45	20.55	19.70	0.03
Ak4	3.64	2.77	2.08	9.82	6.73	6.67	0.01
Akr1b8	15.19	14.50	11.70	31.58	29.56	30.44	0.00
Aldh1a2	31.42	21.82	22.07	168.74	112.97	93.16	0.02
Ampd3	5.34	3.35	2.87	17.05	13.97	12.07	0.00
Apol9a	5.88	3.18	2.98	11.72	7.76	9.34	0.00
Ass1	35.69	35.34	29.90	56.11	97.30	93.35	0.04
AU020206	7.23	2.63	2.42	21.87	12.44	13.55	0.01
Bcl3	2.33	0.94	1.01	11.10	3.96	5.03	0.05
Birc3	3.21	2.57	2.54	9.04	6.96	7.09	0.00
Bst2	85.60	18.06	25.14	116.56	59.82	75.64	0.01
C1qb	3.59	1.49	0.40	9.71	6.81	4.10	0.01
C1s1	28.42	17.80	16.41	112.74	69.23	38.83	0.05
C3	8.36	6.17	4.92	490.62	191.10	180.18	0.05
Car9	2.96	0.88	1.46	5.99	2.43	2.85	0.03
Casp1	3.75	1.12	1.07	5.59	3.76	5.02	0.02
Casp4	2.79	1.55	1.59	7.26	5.33	7.03	0.01
Ccl11	0.70	2.14	0.44	16.51	28.36	35.41	0.02
Ccl2	54.87	137.13	65.60	704.85	839.42	726.01	0.00
Ccl20	0.90	0.94	0.66	37.01	27.24	20.19	0.01
Ccl5	1.31	0.34	0.25	10.10	7.13	7.84	0.00
Ccl7	136.03	301.34	184.27	638.86	704.73	595.16	0.00
Ccl8	2.31	0.78	0.55	16.57	15.18	20.36	0.01
Ccl9	2.59	1.63	0.93	6.97	4.54	4.60	0.01
Cd68	8.84	2.69	1.35	15.77	6.67	6.13	0.01
Cd74	1.23	0.26	0.42	4.46	2.58	4.05	0.01
Cdhr1	1.23	0.69	0.86	5.39	5.50	5.41	0.00
Cdk6	1.26	2.17	2.22	3.02	4.99	5.52	0.01
Cfb	3.72	3.43	4.23	25.38	18.63	13.87	0.02
Cgnl1	8.54	9.92	8.30	24.37	21.12	26.14	0.01
Ch25h	2.05	4.58	2.88	27.96	38.03	30.57	0.00
Col18a1	30.03	21.51	22.70	58.47	53.08	56.07	0.00
Cp	0.78	0.42	0.61	5.12	3.17	2.59	0.02
Csf1r	3.81	1.14	0.59	6.05	1.88	3.16	0.04
Ctsc	1.02	0.93	0.65	4.02	2.27	1.75	0.05
Ctss	1.44	1.29	0.27	8.41	3.52	5.68	0.04
Cxcl1	9.33	11.76	4.81	28.47	30.19	30.81	0.01
Cxcl10	0.62	0.37	0.12	3.49	4.68	5.25	0.01
Cxcl12	58.66	52.39	62.02	186.23	123.52	110.89	0.04
Cxcl16	3.33	1.56	1.87	12.67	10.40	9.63	0.00
Cxcl5	75.89	21.41	18.48	284.31	134.37	141.22	0.02
Cxcl9	0.39	0.06	0.04	3.03	2.49	2.78	0.00
Cyba	23.43	22.26	21.43	57.50	38.50	42.84	0.02
Cyp7b1	6.80	3.38	4.17	21.65	12.65	14.47	0.01
Dram1	11.24	9.14	8.36	44.68	30.10	31.38	0.01
Dtx3l	6.29	1.97	2.39	8.95	5.67	6.75	0.01

Ecscr	7.29	10.00	6.29	35.10	42.80	44.39	0.00
Egln3	1.54	0.96	1.10	7.14	3.25	3.49	0.04
Enpp2	5.08	1.28	1.89	26.84	21.33	39.74	0.02
Errfi1	4.32	21.83	18.10	14.88	34.15	31.82	0.00
F730043M.	0.77	0.57	0.57	4.80	3.68	1.60	0.05
Fabp7	0.47	0.61	0.33	5.13	3.21	1.52	0.05
Fam162a	133.41	75.44	73.28	282.81	149.95	171.24	0.02
Fas	16.29	18.02	20.89	41.58	40.72	38.39	0.01
Fcer1g	3.69	0.75	0.17	7.46	3.96	6.91	0.03
Flrt2	12.27	11.97	10.24	19.14	24.57	31.50	0.04
Flrt3	2.88	3.55	3.46	5.86	7.81	7.98	0.01
Foxs1	2.64	7.70	6.85	8.81	19.39	13.38	0.02
Gas7	0.87	0.28	0.37	3.88	1.84	2.40	0.02
Gbp2	21.50	6.75	16.11	46.76	29.52	32.43	0.01
Gbp3	9.19	1.73	3.45	10.58	7.12	9.33	0.05
Gm12840	3.11	7.31	3.65	11.42	15.17	17.21	0.02
Gm13394	67.14	14.31	54.58	149.90	99.07	91.59	0.02
Gm20547	10.91	4.29	11.93	66.07	33.74	29.51	0.05
Gm26809	14.41	21.06	16.43	40.15	36.52	59.95	0.04
Gm30873	1.41	1.73	1.88	3.98	3.80	3.41	0.01
Gm42793	15.42	11.11	5.84	57.94	29.25	29.59	0.03
Gm43302	0.73	0.24	0.26	3.89	1.97	2.43	0.02
Gm45551	0.91	1.02	1.04	3.28	2.68	3.91	0.01
Gm45774	5.83	2.83	2.83	30.83	11.57	14.43	0.05
Grem2	5.21	9.49	8.93	13.84	26.54	20.19	0.02
Gsdmd	12.88	10.89	12.02	26.57	22.06	24.74	0.00
H2-D1	56.12	33.54	36.91	116.67	80.43	95.24	0.00
H2-K1	46.48	23.85	27.42	129.02	93.23	104.96	0.00
H2-K2	6.70	3.17	4.75	11.52	9.95	7.88	0.02
H2-Q1	3.13	1.54	2.04	6.37	5.42	4.48	0.01
H2-Q4	7.63	2.41	2.84	31.57	23.58	24.39	0.00
H2-T23	12.53	4.06	5.72	26.65	10.63	15.27	0.02
H2-T-ps	10.88	4.36	6.98	24.92	19.84	16.51	0.01
Hist2h2aa2	0.00	0.00	0.00	3.18	3.84	3.76	0.00
Hmga1	4.03	4.18	1.96	9.43	8.73	14.67	0.05
Hmga1b	14.97	14.60	8.72	19.11	34.46	26.54	0.05
Hp	6.65	2.28	4.48	64.49	41.21	23.87	0.04
Hpse	1.34	0.58	0.98	5.12	2.69	3.45	0.02
Icam1	3.36	1.58	2.03	33.85	27.28	25.62	0.00
Ifi203	3.23	0.77	0.88	5.79	2.93	4.26	0.01
Ifi207	2.35	0.56	0.52	3.66	2.29	2.37	0.00
Ifi27l2a	14.71	2.67	4.41	20.27	6.64	12.72	0.02
Ifi44	2.80	2.36	2.57	10.37	6.31	8.25	0.02
Ifi47	1.76	0.64	0.53	8.82	5.27	5.91	0.01
Ifit1	8.77	5.48	7.12	28.21	23.70	28.44	0.00
Ifit3	8.56	6.05	6.32	29.80	21.87	30.14	0.01
Ifit3b	1.32	1.56	1.76	6.11	5.22	6.48	0.00
Igfbp3	6.31	9.87	4.18	19.13	19.54	18.09	0.01
Iigp1	8.88	1.76	1.71	14.89	7.99	9.62	0.00
Il1rl1	36.00	22.26	11.44	67.33	82.79	90.66	0.03
Il4ra	22.43	16.75	12.85	53.65	30.84	33.51	0.02
Irf1	7.00	3.32	3.44	16.50	9.89	12.45	0.01
Irf7	2.37	1.69	2.01	7.45	5.00	6.99	0.01
Irgm2	9.94	3.23	4.77	12.48	10.78	14.12	0.04
Isg15	23.51	8.64	10.86	52.47	37.28	46.52	0.00

Itgb2	1.78	0.46	0.12	7.41	5.11	1.74	0.04
Kalrn	1.03	1.12	0.91	2.61	3.08	2.39	0.00
Kcnf1	0.75	1.85	1.06	4.24	5.09	3.32	0.01
Kcnj15	3.87	1.88	1.06	8.04	5.03	5.92	0.01
Krt19	2.87	2.36	2.02	5.55	5.26	7.77	0.03
Laptm5	4.26	0.76	0.29	10.56	7.49	6.67	0.00
Lbp	9.01	10.03	10.74	50.62	27.37	24.64	0.05
Lrig1	1.90	2.74	2.15	6.06	6.82	5.59	0.00
Lyz1	0.29	0.23	0.06	6.78	3.22	3.37	0.03
Lyz2	48.75	23.69	29.79	64.28	53.18	78.70	0.04
Mgarp	1.70	0.63	0.72	6.86	2.32	2.94	0.05
Micall2	6.60	5.65	4.96	12.75	21.17	17.15	0.03
Mmp13	33.22	11.55	11.14	78.84	32.71	27.42	0.05
Mmp3	6.83	4.53	2.61	76.97	89.03	51.24	0.01
Mmp9	1.24	0.52	0.62	45.85	19.38	29.83	0.03
Mt2	54.85	23.45	25.20	251.40	101.15	122.67	0.04
Ndrg2	2.43	1.41	1.49	5.72	3.18	2.80	0.04
Ndufa4l2	25.27	12.05	20.23	117.69	44.23	59.45	0.05
Nfkb2	14.66	12.68	11.53	28.40	28.98	28.52	0.00
Nfkbia	13.48	12.71	15.27	67.42	56.61	56.62	0.00
Nfkbie	3.33	2.38	2.53	26.41	23.44	23.52	0.00
Ninl	1.47	1.10	1.12	4.94	3.39	3.14	0.01
Npy1r	1.98	1.49	1.38	19.92	9.62	7.56	0.05
Nrg1	3.19	1.76	2.21	4.64	4.82	4.67	0.02
Ntn1	3.10	3.89	4.44	8.52	10.12	11.02	0.00
Oasl2	6.98	1.05	1.44	10.24	6.30	8.58	0.02
Parp10	4.86	1.67	2.11	6.33	4.94	5.05	0.02
Parp14	4.06	0.96	1.33	7.89	4.91	6.30	0.00
Pdgfb	0.77	0.76	0.58	3.59	2.39	1.45	0.04
Pdk1	6.69	3.27	4.03	15.04	7.00	8.03	0.03
Pdlim4	6.02	5.23	4.88	20.37	19.74	17.92	0.00
Phf11d	4.54	0.95	1.52	6.24	4.12	5.37	0.02
Plac8	0.57	0.19	0.29	3.64	1.54	2.05	0.03
Plscr1	5.97	4.51	4.55	11.97	11.08	12.23	0.00
Prelid2	4.51	1.62	2.49	10.78	4.99	5.37	0.03
Psmb10	7.10	4.24	5.41	13.51	11.23	13.22	0.00
Psmb8	4.17	1.38	1.73	9.92	5.91	6.78	0.00
Ptges	11.23	9.51	10.85	26.15	18.98	20.04	0.01
Ptgir	2.23	1.54	1.52	9.07	4.59	6.30	0.02
Rab20	1.02	1.45	1.11	2.32	3.19	2.33	0.01
Rarres2	1.85	1.01	2.19	7.50	3.21	6.96	0.03
Rbp1	1.38	2.07	1.14	4.59	3.64	3.18	0.02
Relb	4.17	3.42	4.27	11.08	11.47	12.82	0.00
Rhbdf2	1.05	0.75	0.57	3.59	3.34	3.57	0.00
Ripk2	9.57	11.09	9.33	22.38	28.60	25.49	0.00
Rnd1	1.90	7.46	6.23	10.01	14.60	12.15	0.00
Rnf150	2.98	1.79	2.15	7.24	4.53	4.62	0.01
Rnf213	2.57	3.39	3.41	11.22	8.80	11.05	0.01
Rps18	3.08	2.02	3.07	21.59	15.50	41.66	0.05
Rrad	3.14	1.94	2.16	9.55	7.25	6.81	0.00
Rtp4	17.02	3.50	4.77	19.96	13.12	16.71	0.05
S1pr3	20.52	15.18	18.79	48.99	46.12	44.93	0.00
Saa3	0.73	0.60	0.32	167.74	88.70	58.75	0.04
Sema4b	2.16	1.30	1.47	3.26	3.27	3.61	0.02
Serpina3f	0.43	0.20	0.31	3.07	2.47	2.33	0.00

Serpina3g	8.75	5.57	4.57	51.84	39.55	36.57	0.00
Serpina3h	1.80	0.84	7.96	12.15	15.72	21.65	0.01
Serpina3i	0.33	0.35	3.74	11.70	8.09	11.35	0.01
Slc16a3	10.90	6.26	5.16	27.63	14.95	14.93	0.02
Slc2a6	1.60	1.04	0.88	4.85	4.86	5.84	0.01
Slc43a2	1.66	1.41	1.38	4.28	3.99	3.34	0.00
Slco2a1	4.70	2.60	3.53	12.95	13.83	16.77	0.01
Slfn2	1.29	0.25	0.03	5.09	2.84	5.19	0.02
Slfn5	4.35	1.70	3.63	8.05	7.10	8.86	0.01
Sod2	10.44	10.00	9.91	30.21	20.31	18.79	0.03
Spp1	774.26	514.29	267.71	2980.36	1315.36	1398.40	0.04
Stx11	1.78	1.27	1.28	3.49	2.59	2.96	0.00
Syt13	0.25	0.50	0.46	2.72	3.46	2.37	0.01
Tapbp	51.29	24.67	32.66	96.09	71.05	83.05	0.00
Tgm2	2.09	1.63	1.36	12.91	7.41	4.71	0.05
Tlr2	5.67	3.79	4.16	18.03	12.93	13.55	0.00
Tmem176a	19.51	14.85	12.08	60.03	45.44	41.46	0.01
Tmem176b	37.75	29.59	24.76	108.20	86.88	79.80	0.00
Tnfaip3	0.49	0.63	0.50	5.81	6.90	6.68	0.00
Tnip1	11.00	8.80	9.60	36.75	28.65	27.12	0.01
Traf3	3.56	3.71	3.54	7.27	7.64	8.20	0.00
Trex1	14.57	6.53	7.92	20.24	18.73	22.12	0.03
Tspan11	0.31	0.46	0.31	3.07	2.08	2.37	0.01
Tyrobp	1.74	1.20	0.35	5.67	2.66	6.19	0.05
Ube2l6	13.12	5.42	8.41	31.00	20.21	20.58	0.01
Uchl1	0.97	1.35	1.57	3.94	4.49	3.04	0.02
Ugt1a7c	3.05	1.27	1.49	12.44	9.52	11.48	0.00
Vcam1	35.50	18.70	23.19	69.15	45.78	47.59	0.00
Vegfc	4.18	5.02	3.40	11.30	11.51	9.36	0.00
Wfdc17	1.21	0.86	0.49	7.90	4.28	2.82	0.04
Xaf1	2.37	1.54	2.10	6.25	4.83	6.45	0.00
Zbp1	1.85	0.35	0.33	5.93	2.75	3.39	0.01

Table S3. Expression levels (RPKM) of mSRGs in FLS from wild type control and TNF transgenin mouse Tg197

Gene Symbol	**WT-1**	**WT-2**	**WT-3**	**TNF_chronic-1**	**TNF_chronic2-1**	**TNF_chronic-3**	**p-value (T-test)**
Alpl	12.29	6.11	5.79	3.52	3.17	1.62	0.05
Apoe	7.02	6.89	7.59	4.39	2.07	1.75	0.02
Arxes1	2.83	4.29	3.57	1.78	1.90	1.54	0.02
Asic3	7.86	4.44	4.87	1.41	2.72	1.13	0.05
Atp12a	25.83	17.95	6.09	10.33	3.29	1.90	0.04
Bmp4	48.76	72.84	94.71	26.13	29.23	20.60	0.04
Bmp5	5.05	3.57	4.78	2.16	1.15	1.30	0.01
C1qtnf3	23.60	42.76	41.55	6.01	3.10	4.08	0.02
Cdsn	26.87	17.64	36.71	5.45	7.24	9.57	0.03
Chrdl1	4.99	4.88	9.15	1.35	1.93	2.57	0.03
Cilp	88.48	81.83	106.37	68.15	35.37	32.82	0.05
Clec14a	5.87	7.01	7.23	2.93	2.80	2.25	0.01
Coch	15.56	26.46	13.60	1.39	4.03	4.53	0.03
Col14a1	18.17	20.73	33.43	8.82	10.51	10.93	0.04
Comp	498.60	507.04	738.05	331.53	166.63	174.56	0.04
Dlk1	3.93	1.70	3.50	0.94	0.76	1.76	0.04
Efs	2.68	3.45	4.57	0.98	1.18	0.79	0.03
Egfl6	36.52	38.04	49.87	18.81	9.80	6.64	0.03
Eln	31.14	65.50	90.96	13.12	12.96	12.64	0.05
Fbln7	3.99	3.66	6.54	1.16	1.00	0.72	0.03
Frzb	5.76	3.77	5.07	3.06	2.11	1.74	0.02
Fzd8	19.65	32.95	44.45	11.01	11.60	11.71	0.05
G0s2	4.79	2.40	3.05	1.73	0.86	0.69	0.02
Galnt9	12.05	10.30	25.24	2.09	0.88	0.76	0.05
Gzmc	26.41	31.02	9.34	9.12	7.95	4.04	0.05
Gzmd	10.25	6.72	6.17	2.73	2.77	1.33	0.02
Id4	16.92	20.48	28.36	7.88	7.98	6.88	0.03
Igfbp2	155.01	598.45	482.09	61.82	187.87	98.79	0.05
Igfbp4	852.70	575.96	963.70	466.50	278.10	293.57	0.03
Kazald1	2.78	4.92	4.72	2.05	1.48	1.71	0.05
Kcnab1	3.39	3.62	2.91	1.46	1.71	1.50	0.00
Kcne4	9.61	10.05	15.01	5.71	2.41	2.19	0.04
Kcnk2	6.39	7.30	10.27	1.95	2.79	2.00	0.02
Lgr5	22.10	31.03	41.78	10.10	6.64	8.42	0.03
Limch1	5.46	5.38	8.18	1.91	2.22	1.56	0.03
Lmcd1	55.52	54.40	71.67	24.30	29.83	21.05	0.02
Mab21l1	3.85	3.74	2.55	1.35	1.13	1.16	0.02
Matn4	2.23	3.90	4.00	0.84	0.35	0.47	0.03
Megf6	12.58	5.53	9.36	5.03	2.59	3.21	0.03
Mest	99.71	79.08	97.33	17.30	16.25	16.69	0.00
Mid2	5.03	6.14	7.89	3.06	2.84	2.37	0.04
Mkx	3.22	6.64	6.24	2.34	2.58	2.74	0.05
Ndnf	7.32	8.08	13.48	3.74	4.59	4.87	0.05
Nell2	5.25	7.90	10.87	2.21	4.21	3.07	0.04
Palmd	4.60	12.05	14.52	1.26	1.29	1.61	0.04
Pappa	30.42	19.35	22.15	15.74	11.32	7.38	0.02
Pcp4l1	23.56	17.03	13.44	3.76	5.13	2.76	0.02
Plagl1	16.46	10.64	13.66	8.64	5.79	5.77	0.01
Ptn	55.52	26.61	35.30	15.50	10.07	18.19	0.04
Ptprd	1.80	2.84	3.13	0.96	1.46	1.24	0.02
Rgs4	30.38	91.74	70.87	15.88	42.24	26.81	0.04
Ror1	4.68	5.40	7.68	3.12	2.88	2.70	0.05

Scg2	7.06	8.13	10.62	1.12	0.23	0.24	0.01
Sfrp1	185.02	145.35	151.88	145.35	55.27	42.47	0.03
Smad9	3.00	2.69	3.50	1.45	1.49	1.34	0.01
Sox9	14.92	8.57	10.01	2.97	4.34	5.05	0.05
Sp7	4.67	1.35	3.54	0.97	0.49	0.48	0.05
Sparcl1	5.36	5.57	4.98	2.54	1.49	2.13	0.01
St8sia2	7.88	6.51	11.49	2.43	3.23	2.71	0.03
Tbxa2r	10.59	9.97	16.09	6.39	5.15	5.18	0.05
Tmtc1	3.94	3.56	4.13	1.14	0.74	0.42	0.00
Tnfrsf21	3.58	4.87	3.49	1.68	2.16	1.38	0.01
Wwc1	2.85	2.92	3.14	1.24	1.80	0.99	0.02

Table S4. Expression level (RPKM) of SAGs and SRGs in human RA-FLS treated with TNF for 8h and 24h

hSAGs

Gene Symbol	RA-FLS-1	RA-FLS-1	TNF_8h-RA FLS-1	TNF_8h-RA FLS-2	TNF_24h-RA FLS-	TNF_24h-RA FLS-2
ASS1	51.99	48.24	92.31	89.13	103.08	98.49
BCL3	11.42	9.05	26.07	21.08	23.30	21.68
BIRC3	6.66	7.28	53.88	60.22	58.86	59.56
BST2	0.22	0.42	60.53	54.83	154.50	160.06
C3	33.16	30.11	72.18	66.41	103.70	101.09
CCL13	0.98	1.20	5.88	5.51	8.76	9.34
CCL5	2.59	3.49	337.86	316.80	1283.76	1270.39
CCL7	0.58	0.27	4.97	5.69	2.60	3.38
CD68	21.63	21.65	38.85	34.15	56.05	52.31
CD74	4.69	4.22	32.38	29.12	69.98	65.60
CDK6	5.63	6.85	20.58	22.36	14.41	14.52
CFB	25.29	23.62	144.69	143.48	318.78	309.82
CH25H	3.51	3.52	52.99	53.27	60.36	57.84
CXCL10	0.07	0.07	128.98	146.64	109.92	112.06
CXCL16	16.27	15.05	31.56	26.87	43.81	42.14
CXCL2	0.39	0.73	19.20	21.05	20.81	18.48
CXCL6	2.90	3.12	75.30	78.37	86.78	90.48
CXCL9	0.00	0.06	40.48	42.75	35.04	33.60
CYP7B1	0.11	0.14	7.89	8.51	4.64	5.19
DTX3L	11.79	13.15	78.19	83.19	70.00	74.08
FAS	22.29	25.44	61.99	62.82	52.21	54.82
GBP4	0.28	0.34	120.40	125.28	66.53	66.59
GSDMD	8.07	6.30	19.00	15.03	18.95	19.03
HLA-B	80.35	68.38	195.55	175.30	362.24	355.11
IFIT3	18.45	20.86	403.07	422.44	325.94	318.66
IRF1	5.03	5.37	60.84	58.08	39.11	40.62
NFKB2	16.86	14.72	62.93	51.96	68.79	66.32
NFKBIA	24.71	25.65	120.33	117.22	117.16	116.95
NFKBIE	7.70	7.61	63.57	54.07	61.76	60.94
NTN1	2.23	1.78	7.45	6.48	16.12	15.69
PARP10	9.82	6.85	38.80	29.47	38.16	36.99
PARP14	10.18	11.61	123.54	131.51	83.02	82.92
PDK1	0.94	1.11	2.75	3.18	2.29	2.39
PLSCR1	6.33	6.78	61.78	66.31	54.62	54.43
PSMB8	27.40	27.16	78.20	73.08	73.20	72.83
PTGES	25.94	23.87	96.64	88.46	121.58	110.50
PTGIR	3.97	3.53	21.05	18.90	21.11	23.26
RAB20	2.20	2.69	7.26	6.24	7.94	7.88
RELB	10.75	10.02	43.39	34.83	49.38	47.29
RHBDF2	1.84	1.67	8.84	8.14	15.79	15.56
RIPK2	10.38	10.91	70.21	68.53	56.05	55.82
RTP4	2.68	1.66	37.42	40.02	43.16	45.85
SLC16A3	10.20	9.01	15.25	12.53	20.73	19.50
SLC2A6	2.38	2.63	37.46	29.95	57.32	56.87
SLC43A2	2.12	1.66	17.79	14.88	5.49	5.34
SLCO2A1	0.09	0.07	3.49	3.77	20.30	20.51
SLFN5	21.61	24.64	48.90	51.80	38.20	39.45
TAPBP	43.94	41.08	123.31	116.11	137.38	131.05
TGM2	7.20	8.56	9.79	7.80	20.64	21.00
TLR2	0.04	0.00	9.69	10.97	18.20	18.62

TNFAIP3	12.04	13.82	176.49	177.00	154.64	145.74
TNIP1	23.64	22.37	80.66	73.53	66.84	63.21
UBE2L6	32.87	31.96	93.97	86.26	117.58	107.46
VCAM1	27.97	30.63	313.15	350.90	165.32	161.07
VEGFC	37.46	37.25	96.67	89.88	91.20	87.04
hSRGs						
COL14A1	87.68	85.87	67.28	63.94	40.04	39.29
KAZALD1	40.50	35.84	21.23	18.48	13.16	13.25
MEST	26.79	27.81	13.43	15.46	7.24	9.05
BMP4	23.36	22.64	2.54	11.66	6.56	5.68
ID4	7.57	7.88	2.69	2.38	2.39	1.60
SMAD9	8.61	8.11	0.95	1.19	1.19	1.10
